# Dose gradient curve: A new tool for evaluating dose gradient

**DOI:** 10.1371/journal.pone.0196664

**Published:** 2018-04-26

**Authors:** KiHoon Sung, Young Eun Choi

**Affiliations:** Department of Radiation Oncology, Gachon University Gil Medical Center, Gachon University School of Medicine, Incheon, Republic of Korea; North Shore Long Island Jewish Health System, UNITED STATES

## Abstract

**Purpose:**

Stereotactic radiotherapy, which delivers an ablative high radiation dose to a target volume for maximum local tumor control, requires a rapid dose fall-off outside the target volume to prevent extensive damage to nearby normal tissue. Currently, there is no tool to comprehensively evaluate the dose gradient near the target volume. We propose the dose gradient curve (DGC) as a new tool to evaluate the quality of a treatment plan with respect to the dose fall-off characteristics.

**Methods:**

The average distance between two isodose surfaces was represented by the dose gradient index (DGI) estimated by a simple equation using the volume and surface area of isodose levels. The surface area was calculated by mesh generation and surface triangulation. The DGC was defined as a plot of the DGI of each dose interval as a function of the dose. Two types of DGCs, *differential* and *cumulative*, were generated. The performance of the DGC was evaluated using stereotactic radiosurgery plans for virtual targets.

**Results:**

Over the range of dose distributions, the dose gradient of each dose interval was well-characterized by the DGC in an easily understandable graph format. Significant changes in the DGC were observed reflecting the differences in planning situations and various prescription doses.

**Conclusions:**

The DGC is a rational method for visualizing the dose gradient as the average distance between two isodose surfaces; the shorter the distance, the steeper the dose gradient. By combining the DGC with the dose-volume histogram (DVH) in a single plot, the DGC can be utilized to evaluate not only the dose gradient but also the target coverage in routine clinical practice.

## Introduction

Stereotactic radiotherapy, often referred to as stereotactic radiosurgery (SRS) or stereotactic ablative radiotherapy (SABR), is a highly focused radiotherapy technique that delivers an intense radiation dose concentrated on a tumor, while limiting the dose to the surrounding normal tissues. SRS or SABR plans should be evaluated based on the quality of target coverage to maximize the dose to the target, and on the steepness of the dose gradient outside the target volume to minimize the dose to organ-at-risks (OARs). A dose-volume histogram (DVH) is most commonly used as a plan evaluation tool to achieve these two objectives [1]. To determine the quality of target coverage, various conformity indices have been introduced and widely used as complementary tools [2–10]. However, there have been limited studies evaluating the dose gradient of the dose distribution.

Dose fall-off characteristics near the target volume can be evaluated by visual inspection of two-dimensional isodose distributions, section by section. It is possible to visualize cross sectional dose profile using dosimetry software, but objective measurement of the dose gradient is nearly impossible. The gradient index (GI), defined as the ratio of the volume of half the prescription isodose to that of the prescription isodose, has been proposed as a simple dose gradient measurement tool [11]. The ratio of 50% prescription isodose volume to the planning target volume (PTV), R_50%_, has been widely adopted as a benchmark for assessing the dose gradient beyond the PTV extending into normal tissue structures [12, 13]. Although the GI and R_50%_ have allowed quantitative analysis of the dose gradient and comparison of competing plans on the basis of these scores, the complexity of the dose profile over the range of dose distribution cannot be taken into account. Furthermore, the current volume-based indices are highly dependent on target volume and shape, and would provide misleading results especially when examining small target volumes or complex target shapes [7].

We propose the dose gradient curve (DGC) as a distance-based dose gradient evaluation tool which is independent of target volume and shape to complement the DVH and/or the conformity index. The dose gradient is represented as a form of a dose gradient index (DGI), the average distance between two isodose surfaces; the shorter the distance, the steeper the dose gradient. The DGC is defined as a plot of DGI as a function of the dose over the range of dose distributions. Two types of DGCs, *differential* and *cumulative*, are available. The performance of DGC was systemically investigated with virtual structures.

## Methods

### Dose gradient index (DGI)

**Fig 1:**
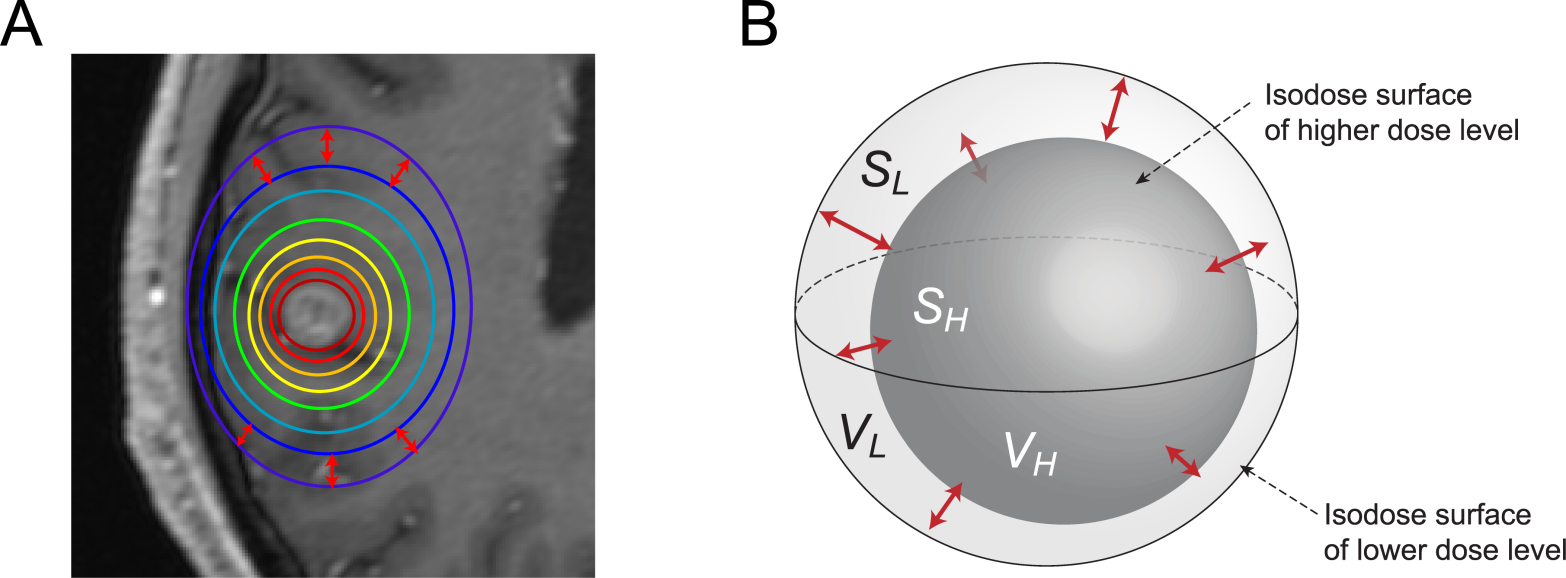
(A) Two-dimensional dose distribution displayed using isodose lines on an axial CT-slice. (B) Three-dimensional illustration of two isodose surfaces. Double-headed arrows indicate the distances between two isodose surfaces. The volume and surface area of isodose levels were used to estimate the average distance between two isodose surfaces. *V* and *S* represent the volume and surface area of an isodose level, and the subscripts *L* and *H* represent the lower and higher doses.

The DGI measures the average distance between two isodose surfaces. The isodose surface is defined as the uniform dose contour receiving a certain dose. The volume and surface area of each isodose level are used to estimate the average distance between two isodose surfaces (Fig 1). The isodose surfaces never intersect each other, and the isodose volume of the lower dose level is always larger than that of the higher dose level. Therefore, an estimation can be made by a simple equation, and the DGI can be defined as:

$$DGI=\frac{V_{L}-V_{H}}{\frac{1}{2}\left(S_{L}+S_{H}\right)},$$ (1)

where *V* and *S* represent the volume and surface area of an isodose level, and the subscripts *L* and *H* represent the lower and higher doses (Fig 1B).

**Fig 2:**
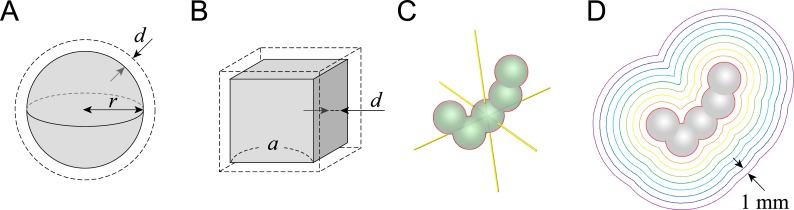
Geometric objects for verification of the dose gradient index (DGI) calculation. (A) A sphere and its uniform expansion with a spacing *d*, (B) a cube and its uniform expansion with a spacing *d*, (C) an irregular shaped structure, and (D) a multi-layer structure at regular intervals (1 mm), produced by uniform expansion of the structure described in (C).

The accuracy of the DGI in estimating the average distance between two isodose surfaces was verified mathematically using simple geometric objects: a sphere and a cube. Each of these structures was expanded uniformly by a distance *d* to produce a double-layer structure with uniform spacing (Fig 2A and 2B). The DGIs obtained from these objects were compared with the actual value, *d* (DGI_sphere_ = DGI_cube_ = *d*).

To verify the clinical applicability of the DGI, an irregularly shaped structure was examined (Fig 2C). A complex structure 2.5 cm^3^ in volume with a concave and convex surface was created using the Eclipse treatment planning system (ver. 13.0; Varian, Palo Alto, CA, USA). This structure was uniformly expanded to generate a multi-layer structure at regular intervals of 1 mm (Fig 2D). The DGI of each interval was calculated, and compared to its actual value of 1 mm. The DGIs computed for every second layer and every third layer, were compared to the actual intervals of 2 mm and 3 mm, respectively.

### Surface area calculation

In this study, surface area calculations were based on a mesh generation algorithm described by Persson and Strang [14]. All geometric analysis was performed with R Statistical Software (version 3.3.2; R Foundation for Statistical Computing, Vienna, Austria). In the R programming environment, the R geometry package (http://geometry.r-forge.r-project.org/) provides high-quality mesh generation and surface triangulation. Using three-dimensional data points representing the isodose surface, the volume of the isodose contour and its surface area were calculated (S1 Code).

### Differential dose gradient curve

**Fig 3:**
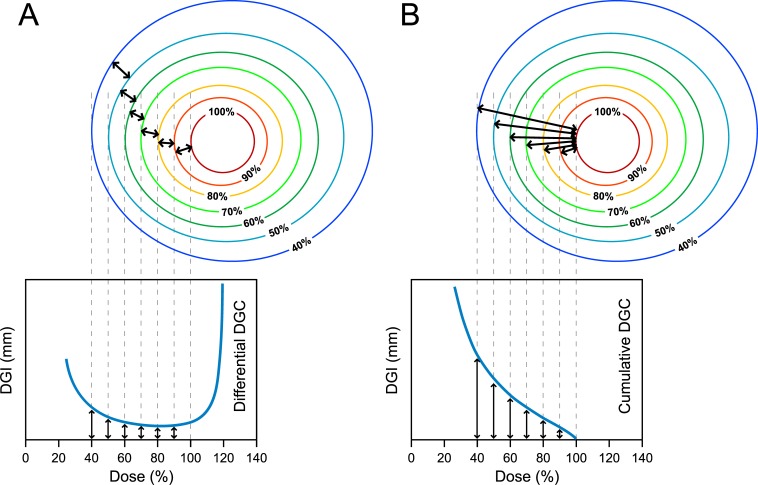
Schematic representation of the basic concept of the dose gradient curves (DGC). (A) The differential DGC and (B) the cumulative DGC.

The *differential* DGI (dDGI) was defined as the DGI of each dose interval, and the dDGI for dose *i* (Gy or %) was calculated as:

$$dDGI_{i}=\frac{V_{i}-V_{i+d}}{\frac{1}{2}(S_{i}+S_{i+d})},$$ (2A)

where *V* and *S* represent the volume and surface area of an isodose level, and *d* is the calculation interval. The *differential* DGC (dDGC) is a plot of the dDGI as a function of the dose (Fig 3A). From the maximum value of the dose distribution to the dose where the isodose surface intersects the body surface (cropped 3 mm from the skin surface), the dDGC shows the dose gradient of each dose interval by the distance value at a millimeter (mm) scale.

### Cumulative dose gradient curve

The *cumulative* DGC (cDGC) is a plot of the *cumulative* DGI (cDGI) and is generated by summing the dDGI values from the reference dose to the desired dose (Fig 3B). The cDGI for the desired dose *i* is defined as:

$$cDGI_{i}=\sum_{j=i}^{D_{0}-d}dDGI_{j},\;cDGI_{D_{0}}=0,$$ (2B)

where *d* is the calculation interval and *D*_0_ represents the reference dose. The cDGI of the reference dose is set to zero (cDGI_*D*0_ = 0), and then every point plotted in the cDGC indicates the average distance from the reference isodose to each isodose surface. By default, the prescription dose (100% isodose) is used as the reference dose. The minimum dose covering 100% of the target volume (D_100%_) is also used as the reference dose, and then the cDGC originates from the point representing 100% target coverage. The plotting range of the cDGC is from the reference dose to the dose where the isodose surface intersects the body surface (cropped 3 mm from the skin surface).

### Performance evaluations of the DGC with SRS plans

The performances of the DGCs were evaluated with a virtual structure in various planning situations. A solitary intracranial spherical target of 3 cm diameter was generated in contouring software. The SRS plan for the virtual target was produced using Dynamic Arc (DA), and the prescription dose was 15 Gy in a single fraction.

The dosimetric influence of the number of arcs with respect to the dose gradient was studied using two virtual targets of 1 cm diameter; one was on the peripheral part of the brain, and the other was near the brainstem. For each of these targets, single fraction SRS plans (24 Gy) were generated using 1, 2, 3, 4, 5, and 7 arcs.

### Radiotherapy planning and data extractions

Structure delineation and generation of SRS plans were performed with the Eclipse treatment planning system using a calculation grid size of 1 mm, and each isodose level was converted to a structure volume. All plans were designed to be delivered on Novalis Tx linear accelerator consisting of high-definition multileaf collimators (HD-MLCs: 2.5 mm leaf width at isocenter). Three-dimensional coordinate data of all isodose structures were exported to the R environment using the Mirada RTx (version 1.6.2.2, Mirada Medical, Oxford, UK).

## Results

Mathematical verification of the DGI was performed using simple geometric objects. For a sphere, as shown in Fig 2A, Eq (1) can be solved to produce the DGI of a sphere (DGI_sphere_) as:

$$DGI_{sphere}=d\times \left\{\frac{\left(r+d\right)+\frac{d^{2}}{3r}}{\left(r+d\right)+\frac{d^{2}}{2r}}\right\},$$ (3A)

where *r* is the radius of the sphere, and *d* is the distance between the spheres. In the same way, the DGI of a cube (DGI_cube_) can be expressed as:

$$DGI_{cube}=d\times \left\{\frac{\left(a+2d\right)+\frac{4d^{2}}{3a}}{\left(a+2d\right)+\frac{4d^{2}}{2a}}\right\},$$ (3B)

in which *a* is the edge length of the cube. The relative error (*e*_*r*_) of the DGI for simple geometric structures can be described as:

$$e_{r}=1-\left\{\frac{\left(r+d\right)+\frac{d^{2}}{3r}}{\left(r+d\right)+\frac{d^{2}}{2r}}\right\}\approx 1-\left\{\frac{\left(a+2d\right)+\frac{4d^{2}}{3a}}{\left(a+2d\right)+\frac{4d^{2}}{2a}}\right\}.$$ (3C)

When 2*r* = *a* = 10 mm, we find that the relative error for *d* = 1 mm is 0.0055 (0.0055 mm), and the relative error for *d* = 0.3 mm is 0.0006 (0.0002 mm).

The accuracy of the DGI was investigated by using the multi-layer structure at 1-mm regular intervals with an irregular surface and shape, as shown in Fig 2D. The calculation results from three different calculation intervals are presented in Table 1. The DGI calculated using adjacent layer pairs ranged from 0.991 to 1.023 mm. The DGI of every second layer ranged from 1.986 to 2.028 mm and that of every third layer ranged from 2.986 to 3.004 mm.

**Table 1:** Dose gradient index (DGI) of the multi-layer structure generated by uniform expansion of an irregularly shaped structure at 1-mm regular intervals.

Layer interval (mm)	Parameters for DGI calculation		DGI of corresponding layer intervals		
	Surface area (mm^2^)	Volume (mm^3^)	1 mm	2 mm	3 mm
0	1477.332	4099.663			
1	1802.676	5778.131	1.023454		
2	2154.435	7781.413	1.012497	2.027526	
3	2522.306	10106.71	0.994409	2.001663	3.003796
4	2920.762	12842.98	1.005416	1.994630	2.991402
5	3345.715	15992.61	1.005229	2.006092	2.985806
6	3789.611	19550.83	0.997355	1.999247	2.992473
7	4261.514	23565.85	0.997381	1.991066	2.985925
8	4768.102	28178.60	1.021692	2.016372	3.003763
9	5286.241	33161.19	0.991131	2.009966	2.999245
10	5829.997	38702.98	0.997063	1.986088	2.999972
11	6411.771	44898.54	1.012200	2.006726	2.991078
12	7006.067	51577.42	0.995523	2.005980	2.996383
13	7627.859	58884.88	0.998701	1.992409	2.999274

Table 2 shows the results of DGI calculation using the SRS plan generated by 3 Dynamic Arc for a virtual target with a 3 cm diameter. A 15 Gy dose in a single fraction was prescribed, and no normalization was performed. With a calculation interval (step size) of 1%, the dDGI ranged from 0.10 to 2.29 mm, and the maximum dDGI outside the target volume was 0.99 mm. The cDGI ranged from 0 mm at the reference dose (prescription dose) to 17.26 mm at the 24% isodose level. The cDGI of the 50% isodose level was 5.98 mm.

**Table 2:** Dose gradient index (DGI) of SRS plan for a virtual target of 3 cm diameter with a prescription dose of 15 Gy in a single fraction. (calculation interval = 1%).

Dose (%)	Dose (Gy)	Surface area (mm^2^)	Volume (mm^3^)	dDGI (mm)	cDGI (mm)
24	3.60	16497.6	165740.9	0.99	17.26
25	3.75	15268.0	150037.0	0.87	16.27
26	3.90	14272.0	137215.8	0.78	15.40
27	4.05	13438.1	126401.9	0.73	14.62
28	4.20	12682.9	116848.6	0.65	13.89
29	4.35	12016.5	108844.4	0.58	13.24
30	4.50	11416.7	102008.5	0.57	12.66
~	~	~	~	~	~
50	7.50	6187.0	44735.6	0.18	5.98
~	~	~	~	~	~
90	13.50	3722.9	21025.7	0.10	1.13
91	13.65	3677.5	20655.6	0.11	1.03
92	13.80	3628.4	20248.4	0.10	0.92
93	13.95	3586.0	19887.5	0.11	0.82
94	14.10	3537.9	19486.2	0.11	0.71
95	14.25	3490.1	19088.6	0.10	0.60
96	14.40	3444.6	18728.2	0.11	0.50
97	14.55	3397.9	18335.1	0.11	0.39
98	14.70	3346.7	17949.4	0.13	0.28
99	14.85	3293.4	17515.5	0.15	0.15
100	15.00	3235.7	17035.3	0.13	0.00
101	15.15	3180.6	16606.7	0.15	
102	15.30	3124.0	16144.7	0.13	
103	15.45	3068.3	15731.0	0.15	
104	15.60	3009.2	15284.3	0.16	
105	15.75	2944.0	14801.0	0.18	
106	15.90	2876.2	14290.8	0.20	
107	16.05	2804.1	13719.9	0.18	
108	16.20	2732.5	13226.9	0.23	
109	16.35	2648.1	12608.6	0.25	
110	16.50	2556.7	11946.2	0.25	
111	16.65	2464.2	11308.0	0.31	
112	16.80	2356.9	10557.6	0.33	
113	16.95	2241.2	9804.0	0.39	
114	17.10	2109.2	8944.8	0.46	
115	17.25	1960.4	8005.2	0.54	
116	17.40	1792.9	6993.9	0.68	
117	17.55	1594.9	5837.9	0.83	
118	17.70	1365.5	4612.7	1.15	
119	17.85	1077.1	3207.2	1.60	
120	18.00	731.1	1763.2	2.29	
121	18.15	341.0	536.3		

SRS, stereotactic radiosurgery; dDGI, differential dose gradient index; cDGI, cumulative dose gradient index.

**Fig 4:**
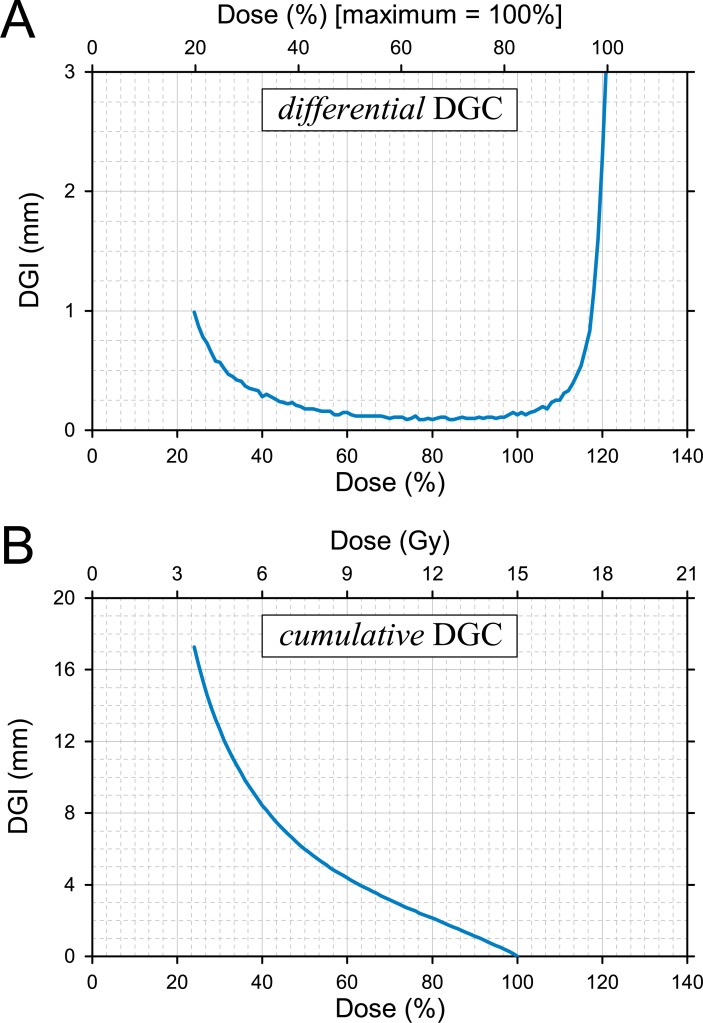
The dose gradient curve. (A) The *differential* dose gradient curve (dDGC) generated by data from Table 2. The dDGC is a plot in which each point represents the average distance between each isodose interval (the *differential* dose gradient index; dDGI); the shorter the distance, the steeper the dose gradient. (B) The corresponding *cumulative* dose gradient curve (cDGC) is a plot of the *cumulative* dose gradient index (cDGI) generated by summing the DGI values from the prescription dose (100% isodose) to each dose. Each point of the cDGC indicates the average distance from the prescription isodose surface to the corresponding isodose level.

**Fig 5:**
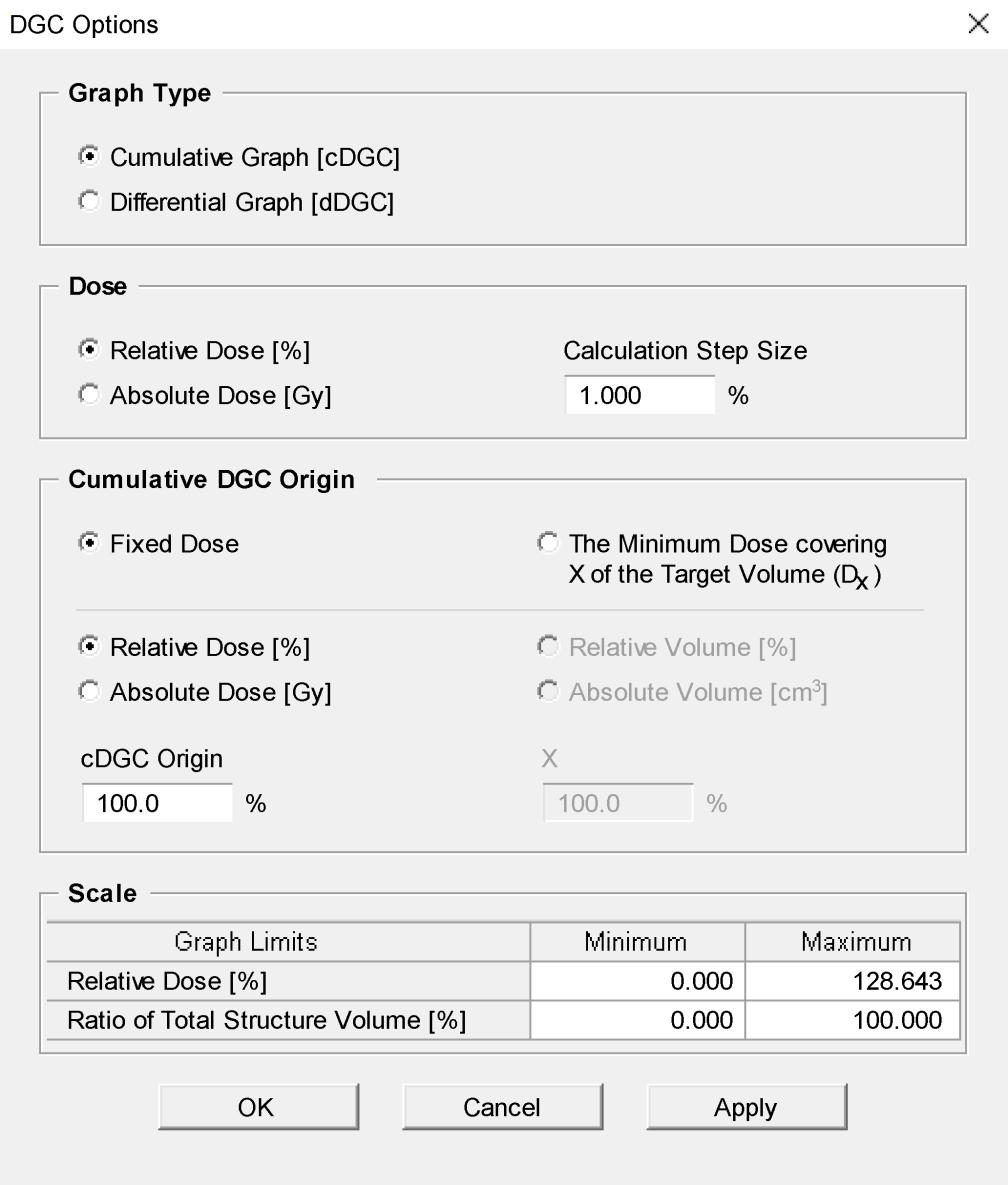
Plot options for the dose gradient curve (DGC).

The data from Table 2 were presented by plotting the DGI against the dose level, as in the DGC proposed in this study (Fig 4). The dose gradient of each dose interval was plotted as a form of the *differential* DGC (dDGC) at a millimeter scale (Fig 4A). From the maximum dose to the 24% isodose level, the dDGC produced a U-shaped curve. The minimum value indicating the steepest dose gradient was observed in the range of 70% - 90% isodose level. As shown in Fig 4B, the *cumulative* DGC (cDGC) was generated. The cDGC demonstrated a characteristic rotated sigmoid shape when plotted with the cDGI on the y-axis versus the isodose level on the x-axis. The cDGC started from the prescription isodose level (cDGI_100%_ = 0) and moved downward from there. All plot options for the DGC are listed in Fig 5.

### The effect of calculation interval

**Fig 6:**
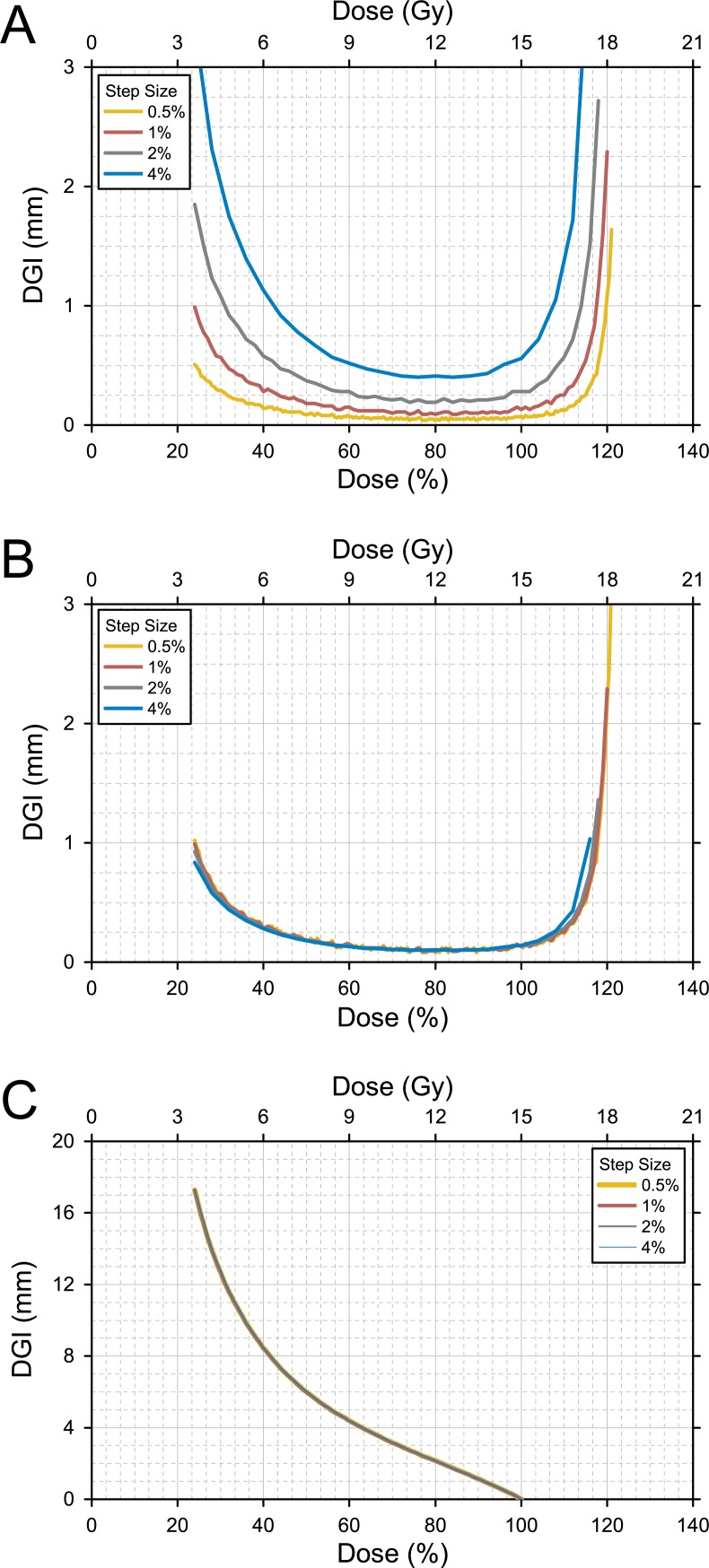
The effect of the calculation interval (step size). (A) The *differential* dose gradient curve (dDGC) for varying step sizes ranging from 0.5% to 4%. The dDGC increased with increasing step size. (B) The *normalized* dDGC showing identical calculation results regardless of the difference in step size. (C) The *cumulative* dose gradient curve (cDGC) for different step sizes. The cDGC is invariant with respect to changes in step size.

The dDGCs for varying calculation intervals (step sizes) were plotted (Fig 6A). The dDGC increased with increasing step size. The dDGC was normalized by dividing it by the step size, and the normalized dDGC showed nearly identical calculation results regardless of the difference in step size (Fig 6B). On the other hand, step size had no effect on the calculation results of the cDGC (Fig 6C).

### Performance evaluations of the DGC with SRS plans

**Fig 7:**
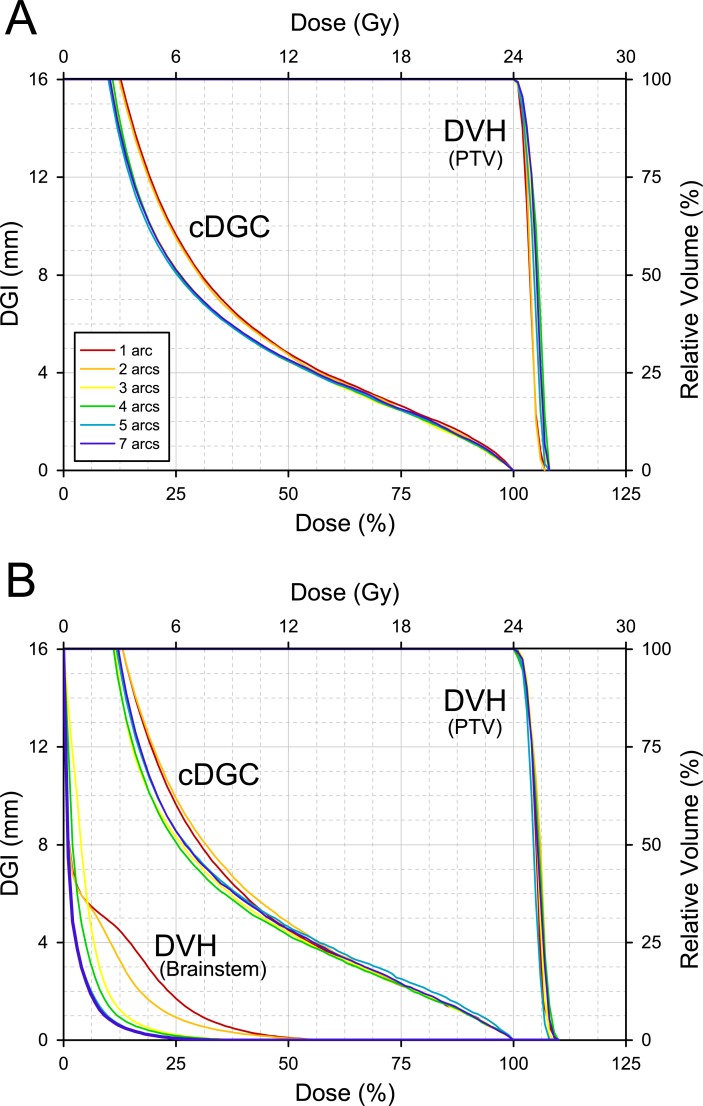
The dosimetric influence of the number of arcs with respect to the dose gradient. The combination of the cDGC and the DVH, which were generated from a virtual target with a 1 cm diameter located (A) on the peripheral part of the brain, and (B) near the brainstem. PTV, planning target volume.

SRS plans for a virtual target of 1 cm diameter were generated using Dynamic Arc (1, 2, 3, 4, 5, and 7 arcs) with varying tumor locations, and all plans were normalized to deliver the prescription dose (24 Gy) to cover 100% of the target volume (V_100%_ = 100%). The combined plot demonstrated the influence of varying the number of arcs on the cDGC and the DVH (Fig 7). Fig 7A shows the cDGC and the DVH generated by SRS plans for a tumor located in the parietal lobe without any OARs near the target volume. The cDGC moved downward with an increase in the number of arcs from 1 to 3, and no further changes were observed with increasing the number of arcs from 3 to 7. The cDGC and the DVH of SRS plans for the tumor near the brainstem are shown in Fig 7B. The doses to the brainstem were consistently reduced as the number of arcs increased, but there was no relation between the number of arcs and the steepness of the dose.

**Fig 8:**
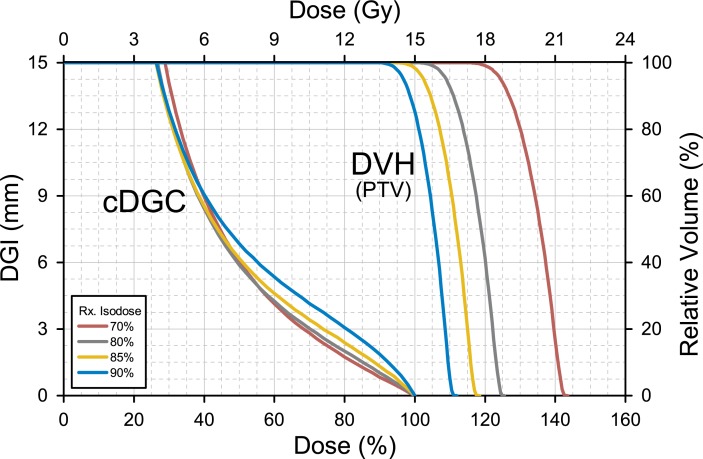
The *cumulative* dose gradient curve (cDGC) combined with a dose-volume histogram (DVH). The combined plot illustrates differences in dose gradient (cDGC) and target coverage (DVH) when doses are prescribed (Rx) at 70%, 80%, 85%, and 90% isodose levels in stereotactic radiosurgery (SRS) plans for a virtual target of 3 cm in diameter. PTV, planning target volume.

Fig 8 shows the cDGC plots for SRS plans combined with the DVH. The SRS plan from Table 2 was normalized to prescribe doses to 70%, 80%, 85%, and 90% isodose levels. As shown in Fig 8, the cDGC changed with the prescription (Rx) isodose levels not only in its values but also in its shape. A strongly concave shape near the prescription dose and the higher levels of the cDGI were observed when a dose of 15 Gy was prescribed to the 90% isodose surface. The shape of the cDGC was gradually changed to a convex shape with a decrease in the prescription isodose, and the lowest level of cDGC near the prescription dose was observed with a prescription isodose of 70%.

## Discussion

The DGC is a rational visualization method for evaluating the plan with respect to the steepness of the dose profile (Fig 3). The dose gradient is representable in an easily understandable format by converting three-dimensional dose distributions to one-dimensional distances between two isodose surfaces. The *differential* DGC provides dose gradient characteristics over the range of dose distributions regardless of the target volume (Fig 4A). In comparison, the *cumulative* DGC demonstrates fall-off characteristics outside the reference dose (Fig 4B). The cDGC was originated from the reference dose level with a value of zero (cDGI_reference_ = 0) in such a way that the cDGC could be plotted in a more easily comparable format (Fig 4B). The dDGC and cDGC were equally effective in evaluating the dose gradient, but the dDGC was superior for profiling the dose distribution, whereas the cDGC was superior for comparing rival plans.

The DGI was calculated based on an estimation of the average distance using the volume and surface area as shown in Eq (1). The error of the estimation was evaluated mathematically and by simulation of an irregular shaped structure (Fig 2). Mathematically, the error of the DGI in estimating the actual distance of 1 mm was only 0.0055 mm at a tumor diameter of 10 mm (Eq 3C). In simulation tests using a multi-layer structure with a regular interval of 1 mm, the error of the estimation ranged from -0.009 to 0.023 mm. This error decreased with decreasing distance between isodose surfaces, and the relative error for 0.3-mm distance was 0.0006 (0.0002 mm). The DGI was calculated differentially with a fine step size and usually ranged from 0.1 to 1.0 mm (Fig 4A). Given the clinical situation, the error in the DGI will be less than a value on the order of 10^−2^ mm.

The DGC can be generated for various calculation intervals (step sizes). As step size refers to the interval between isodose levels used in calculating the dDGC, the average distance increases with increasing step size (Fig 6A). The dDGC could be normalized by dividing it by step size, and the normalized dDGC provided an adjusted value in a common scale. The estimated value will be close to the actual value when the gap between two isodose surfaces is very close, and a step size not greater than 1% or 1 Gy would be recommended in generating the DGC.

The dose-volume histogram (DVH) has been accepted as an essential tool for plan evaluation and can be generated by the treatment planning system. The incorporation of the DGC into the treatment planning system could enable immediate calculation and visualization of the DGC just as for the DVH, which uses the same coordinate system as the DGC. By virtue of the combined plot of the DGC and the DVH, the dose gradient as well as the target coverage can be evaluated in a single plot with double y-axis.

Increasing the number of arcs provides additional flexibility when tailoring the dose distribution. A key question is what number of arcs should be used to generate the maximum dose gradient for an SRS/SABR plan. In this study, the dosimetric influence of the number of arcs with regard to dose fall-off characteristics was studied by the use of the cDGC. Without any consideration of OAR sparing near the target volume, the cDGC showed that the maximum steepness of the dose gradient was obtained using 3 dynamic arcs (Fig 7A). The dose steepness was not further improved by increasing the number of arcs above 3 arcs. With regard to the tumor close to the brainstem, increasing the number of arcs consistently reduced the brainstem dose (Fig 7B). However, the steepest dose gradient was observed in the cDGC of the SRS plan using 4 dynamic arcs, above which the dose gradient became worse. This result could be interpreted as stating that the maximum dose constraint to the brainstem interrupted the uniformity of the dose gradient around the target, and the number of arcs above 4 arcs possibly resulted in increased dose spillage on the opposite side of the brainstem. Using the DGC in addition to the DVH, it was possible to detect another aspect of the dose distribution. In this way, the DGC can be used to evaluate the beam characteristics according to varying treatment machine, the leaf width of a multi-leaf collimator, the dose rate, and treatment technique.

The SRS/SABR dose should be prescribed to the target volume between the 60% and the 90% isodose levels of the total dose (not the prescription dose), and a 50% higher dose prescribed to the isocenter is acceptable [15, 16]. The dose gradient outside the target volume should be optimized to achieve the fall-off recommendations [9]. The two types of DGCs can be used to determine the optimal prescription isodose. For example, it is possible to analyze the dose distribution of an SRS plan (the data from Table 2) by using the dDGC such that the steepest dose gradient is observed in the range of 70% - 90% isodose level (Fig 4A). For those ranges of prescription isodose levels, the cDGCs and the DVH were plotted in a single graph (Fig 8). Of the cDGCs, a red line was drawn below the other cDGCs in the range of 50% - 100%, demonstrating that the steepest dose gradient near the prescription dose could be generated when a dose was prescribed to the 70% isodose surface. However, the relative position of the red line shifted to the top in the range of <40% isodose levels. In contrast, when 15 Gy was prescribed to the 90% isodose level, the cDGC (blue line) was drawn and was always on top. Furthermore, the DVH for this situation indicated insufficient PTV coverage. Consequently, the 80% isodose level (gray line) was determined to be an optimal prescription isodose level in terms of dose steepness. Similar results were demonstrated by a previous study that proposed the dose-dropping speed (DDS) to evaluate the SRS plan quality with regard to the optimal prescription isodose levels [17]. The DDS was defined as the greater decay coefficient in a double exponential decay fit of the dose drop-off outside the PTV, and the highest DDS values were observed for the prescription at 60% or 70% isodose level, representing the best normal tissue sparing.

Various indices such as the Gradient Index (GI), the Conformity Gradient Index (CGI), and R_50%_ have been used for evaluating the dose fall-off outside the target volume [10, 11]. However, these indices were based on limited information from two isodose levels (50% and 100%), and could not reflect all aspects of the dose distribution, even with the use of R_30%_ and R_60%_. For example, when the cDGI of 50% (cDGI_50%_) was the same, the actual dose gradient outside the prescription isodose volume could vary, as shown in Fig 8. Given this situation, the DGC could provide comprehensive way to assess a treatment plan in terms of dose fall-off characteristics.

**Fig 9:**
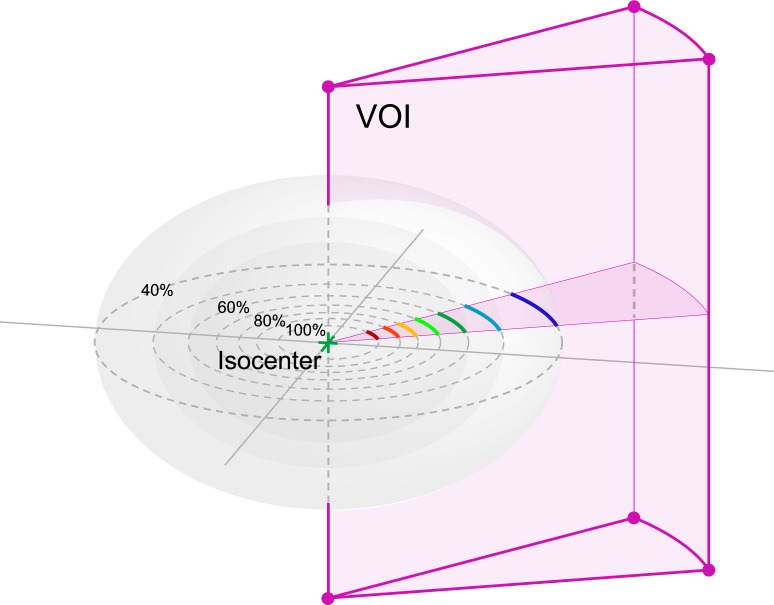
The cylindrical sector-like Volume-of-Interest (VOI), with its axis passing through the planning isocenter. Using the VOI, the DGC would provide the information about the dose gradient in a particular direction.

There are several limitations associated with this study. The DGC did not provide any directional information but provided the average distance between two isodose surfaces. However, the DGC can be modified to represent the dose gradient only in a particular direction by means of the Volume-of-Interest (VOI). We can define the VOI enclosed by two radial planes to form a cylindrical sector-like volume with its axis passing through the isocenter. This allows to narrow down the range of calculation in a specified direction, as shown in Fig 9. The validity of the DGC using the VOI is still under investigation, and will be the subject of future reports. Although the clinical applicability of the DGC was examined by different targets in different situations, the SRS targets used in this study were virtually designed structures. Moreover, only brainstem was used to verify the influence of the dose gradient in normal tissue sparing. To establish the clinical significance of the DGC, further research is necessary to determine whether the DGC parameters will translate into dosimetric parameters of various normal tissues in different clinical scenarios.

## Conclusions

In this study, we proposed and assessed the DGC as a new tool to evaluate the quality of a treatment plan. The DGC rendered it possible to evaluate the dose gradient comprehensively over the range of dose distributions and to compare rival plans objectively. Despite certain limitations, our results demonstrate that the DGC, as a complementary tool, provides useful information that cannot be obtained by any other indices or display tools. Moreover, in combination with the DVH in a single plot at the same dose scale, the DGC can be utilized to evaluate not only the dose gradient but also the target coverage in routine clinical practice.

## Supporting information

S1 Code R code for calculating the DGI parameters including volume and surface area. (PDF)
