# Supplementary material for: Dose gradient curve: A new tool for evaluating dose gradient
Source: PLoS One. 2018 Apr 26;13(4):e0196664. doi: 10.1371/journal.pone.0196664 (PMC5919624; doi:10.1371/journal.pone.0196664)
Supplement: S1 Code — (PDF) [file pone.0196664.s001.pdf]

```r
# R code for calculating the Dose Gradient Index (DGI) parameters
# Authors : Sung, KiHoon, MD (novalis@gilhospital.com) and Choi, Young Eun, PhD

# Set working directory containing the 3D coordinate data
setwd("C:/...")

# Load libraries
require(geometry)
require(rgl)
require(dplyr)

# Read in the CSV data and store it in a variable
# Prepare your csv of 3D coordinates of isodose levels
# The first row should include header name of "x, y, z, dose"
files = list.files(pattern = "csv")
xyzd = do.call(rbind,lapply(files, read.csv, header=TRUE))

factor.dose = as.factor(xyzd$dose)    # Dose interval -> Factor
group = levels(factor.dose)           # Dose interval group
len.group = length(group)             # No. of Dose interval

# Set table for DGI parameters
dgi.para = matrix(ncol = 2, nrow = len.group)
colnames(dgi.para) = c("Area", "Volume")

# Calculation of surface area and volume of each isodose level
for(i in 1:len.group) {
  xyzd.sub = xyzd[xyzd$dose==group[i],c(1:3)]            # Grouping by Dose interval
  data.calc = convhulln(xyzd.sub, "FA")                  # Mesh generation and surface tesselation
  dgi.para[i,] = c(data.calc$area, data.calc$vol)        # Matrix formation
}

# Dose interval to rowname (x axis)
rownames(dgi.para) = group

# Write a CSV file containing DGI parameters (Dose, Area, Volume) to the working directory
write.csv(dgi.para, file="dgi_para.csv")
```
